# An Umbrella Review of Determinants of Digital Health Tool Implementation in Chronic Disease Prevention and Management in Clinical Practice Settings

**DOI:** 10.5888/pcd23.260131

**Published:** 2026-11-05

**Authors:** Amanda Gilbert, Rachel M. Scrivano, Angeline Gacad, Loni Parrish, Oliver T. Nguyen, Kea Turner, Willi L. Tarver, Pallavi Jonnalagadda, Dennis Tsagli, Oghosa Evbuomwan, Bowen Wu, Maura M. Kepper

**Affiliations:** 1 Prevention Research Center at Bursky School of Public Health, Washington University in St. Louis, Missouri; 2 Washington University in St. Louis, Brown School of Social Work, Missouri; 3 Department of Population Health, University of Kansas, Kansas City, Missouri; 4 Department of Industrial and Systems Engineering, University of Wisconsin at Madison, Wisconsin; 5 School of Nursing, The University of North Carolina Chapel Hill, Chapel Hill, North Carolina; 6 Division of Cancer Prevention and Control, Department of Internal Medicine, The Ohio State University College of Medicine, Columbus, Ohio; 7 Department of Biomedical Informatics, The Ohio State University College of Medicine, Columbus, Ohio

## Abstract

**Introduction:**

Chronic diseases account for most illness, death, and health care spending globally and in the US. Digital health tools (DHTs) (eg, telemedicine, remote monitoring) can improve chronic disease prevention and management. However, population-level impact depends on successful implementation in clinical care. Although reviews describe determinants of DHT implementation, evidence is fragmented, and no comprehensive qualitative synthesis has focused exclusively on chronic disease prevention and management.

**Methods:**

We conducted a subanalysis of an umbrella review that examined determinants of DHT implementation in clinical practice settings. By using established scoping review methods and PRIOR (Preferred Reporting Items for Overview of Reviews) reporting guidelines, we searched PubMed, Scopus, and CINAHL for English-language reviews published from January 2013 through July 2025. We included reviews that focused on determinants of DHT implementation in clinical care and identified 15 reviews focused on chronic disease prevention and management. We extracted qualitative data on barriers and facilitators and synthesized the data by using the Consolidated Framework for Implementation Research (CFIR).

**Results:**

Key innovation-level determinants included relative advantage, design, and strength of the evidence base, which frequently acted as barriers due to concerns about clinical effectiveness, workflow fit, and data reliability. Most implementer-level barriers related to limited opportunity and capability, including time constraints and low digital literacy among health care team users. Inner setting determinants included access to knowledge and information, available resources, structural characteristics, and compatibility with existing workflows as both barriers and facilitators. Outer setting determinants included a lack of policies and regulations and limited partnerships for supporting DHT implementation.

**Conclusion:**

Implementation of DHTs for chronic disease prevention and management is shaped by multilevel determinants across CFIR domains. Addressing evidence gaps, investing in training and infrastructure, aligning tools with clinical workflows, and strengthening policy and partnership environments are critical to optimizing implementation. Findings provide actionable insights to guide future research and implementation strategies aimed at improving DHT implementation.

**Summary**

**What is already known on this topic?**

Digital health tools (DHTs) in clinical care can improve chronic disease prevention and management by supporting self-management, adherence, monitoring, and patient–provider communication.

**What is added by this report?**

This umbrella review synthesized 15 reviews and used an implementation science determinants framework to identify multilevel implementation determinants. Findings underscore the need for fit with workflow, supportive infrastructure including electronic health record (EHR) integration, and organizational policies to incentivize adoption and use to support chronic disease prevention and management.

**What are the implications for public health practice?**

Practitioners should select and design DHTs that fit clinical workflows, invest in training and technical support, strengthen EHR and infrastructure integration, and build clear policy and insurance reimbursement frameworks. Implementation strategies should address barriers to improve adoption, sustainability, and equitable use of DHTs for chronic disease prevention and management.

## Introduction

The incidence and prevalence of chronic diseases (eg, cardiovascular disease, diabetes, cancer) are increasing in the US and globally, contributing to substantial health and economic burdens (1). They account for an estimated 74% of global deaths (1) and more than 90% of the $4.9 trillion in annual US health care spending, while contributing to reduced quality of life, disability, and lost productivity (2,3).

Digital health tools (DHTs), including telemedicine and telehealth, mobile health applications, electronic health records (EHRs), wearable devices, remote monitoring systems, patient portals, and decision-support tools (4,5), support the prevention and management of chronic diseases by enhancing self-management, improving adherence to treatment regimens, facilitating early detection and monitoring, and supporting communication between patients and providers (6–8). Meta-analyses report benefits across chronic disease outcomes, including improved glycemic control in patients with type 2 diabetes (9), blood pressure reduction (10), and increased physical activity (11), in addition to enhanced patient satisfaction (6).

As the pace of technological innovation increases and the prevalence of chronic diseases grows, DHTs will expand in scope and influence and their demand will be amplified (2). Optimizing the implementation of DHTs is critical to strengthen health systems and improve chronic disease prevention and management (12–14). Moreover, building on known determinants of DHT implementation for chronic disease, such as data privacy, patient preference for in-person consultation, fit within workflows, trust the provider has in the DHTs, and burden (eg, time to learn DHTs) can improve DHT adoption, use, and sustainability in clinical care settings (12,13,15,16). To date, no qualitative synthesis or umbrella review that uses an implementation science framework has been conducted on determinants of DHT implementation for chronic disease prevention and management specific to clinical care settings. Synthesizing these determinants across DHT type and context helps identify barriers and facilitators across DHTs to create tailored implementation strategies to enhance DHT impact on chronic disease prevention and management (17).

In this subanalysis of an umbrella review, we aimed to identify, categorize, and describe common barriers and facilitators from reviews on DHTs for chronic disease prevention and management.

## Methods

We conducted a subanalysis of an umbrella review (hereinafter, parent review) that examined common determinants of DHT implementation in clinical settings. We collected data from a subset of reviews on chronic disease prevention and management. This subanalysis and the parent review followed established methods for reporting guidelines in the Preferred Reporting Items for Overview of Reviews (PRIOR) (18). The review protocol is registered with PROSPERO (www.crd.york.ac.uk/PROSPERO/view/CRD42024501642). An umbrella review is a high-level synthesis of existing reviews and is an appropriate method given the increasingly prominent role of DHTs in clinical care, the substantial body of evidence on use of DHT in clinical settings, and the importance of chronic disease prevention and management (19).

### Data sources

The parent review search strategy was developed with the assistance of a reference librarian. Three databases were searched: PubMed, Scopus, and CINAHL. The initial search captured reviews published from September 29, 2013, through November 6, 2023. The search was updated to include reviews published through July 10, 2025. An approximate 10-year time frame aligns with the growth of DHTs in health care (20). The search terms included both medical subject headings (MeSH) and free-text terms related to digital tools, clinical care settings, and implementation (Table 1).

**Table 1:** Inclusion and Exclusion Criteria, Subanalysis of an Umbrella Review of Determinants of Digital Health Tool Implementation in Chronic Disease Prevention and Management in Clinical Practice Settings (N = 15), 2013–2025

Criterion	Include	Exclude
**Year published **	2013 to 2025	Before 2013
**Language**	English	Non-English
**Country**	All	No exclusions
**Study type**	Systematic review/scoping review/literature review/narrative review/rapid review/meta-analysis/literature analysis	All other study types: observational studies (eg, cohort, case-control, cross-sectional, case-crossover, ecologic, case series, case reports); qualitative studies; commentary or debate papers; conference abstracts; protocol papers (with no reported outcomes)
**Intervention targets**	Clinical care settings (eg, ambulatory care facilities, hospital outpatient clinic, safety net provider, academic medical centers)	Non-clinical care settings (eg, home, school, long-term care facilities, imaging and radiology centers, rehab centers, mental health facilities)
**Intervention type**	Digital health tool including EHRs, AI, computerized decision support systems, telemedicine, smartphone applications, websites, wearable devices, text messaging, health monitoring systems, and telephone support	Does not include HIT or includes technologies that are outside our scope (eg, blockchain technologies)
**Area of focus**	Primarily addresses barriers (eg, challenges, hinderances) and facilitators (eg, advantages, enablers) to implementing digital health tools	Does not address barriers and facilitators to implementing digital health or presents them in passing (eg, not one of the review aims)
**Intended users/adopters of the digital health tool**	Health care providers (eg, doctors, nurses, nurse practitioners, social workers, dieticians, physical therapists, exercise physiologists, medical assistants, clinical research assistants, community health workers)	Non–health care providers (eg, patients, caregivers, medical students, administrators)

Abbreviations: AI, artificial intelligence; EHR, electronic health records; HIT, health information technology.

### Study selection

Reviews must have been published in English; explicitly identified determinants to implementing digital tools in clinical care settings (eg, inpatient hospital setting, outpatient routine care, outpatient emergency care); published from 2013 through 2025; and available in full-text (Appendix).

The search yielded 5,839 reviews (initial search: 4,180 reviews; updated search: 1,659 reviews). Reviews were imported into Covidence, and duplicates were removed. Dual-blinded independent screening was conducted for title, abstract, and full-text review (A.S.G., A.E.G., L.P., M.K., D.T., O.E.). Reviewers held consensus meetings throughout the screening process to resolve conflicts and achieve agreement on inclusion and exclusion of reviews. The PRISMA (Preferred Reporting Items for Systematic Reviews and Meta-Analyses) flow diagram (Figure) shows the number of reviews at each stage.

**Figure:**
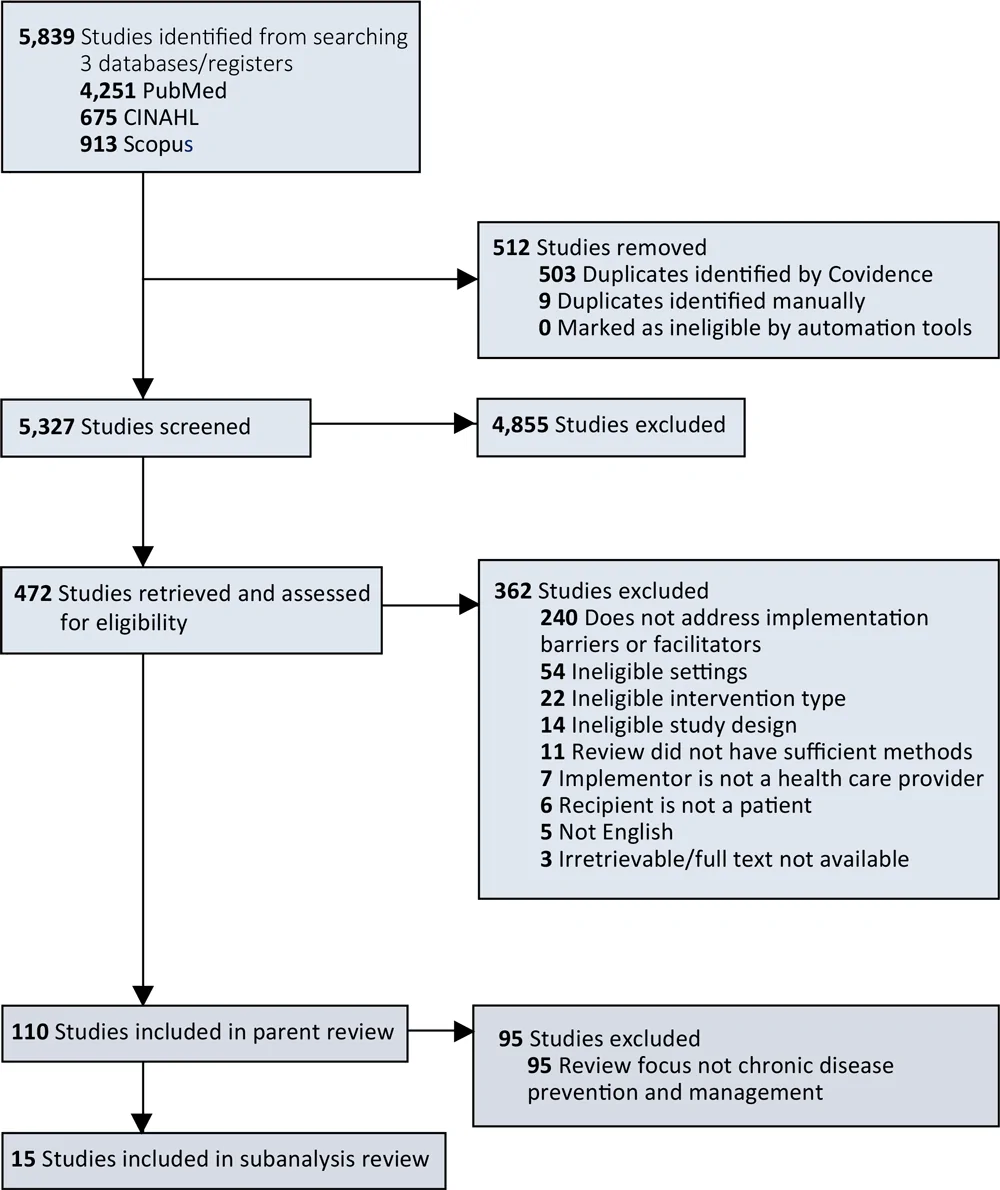
PRISMA overview of selection of studies for inclusion and exclusion, subanalysis of umbrella review of determinants of digital health tool implementation in chronic disease prevention and management in clinical practice settings, 2013–2025.

### Data extraction

Due to the large sample of reviews in the parent review (n = 110), extracting and summarizing qualitative data to meaningfully categorize and describe common determinants of DHT implementation were not feasible. The study team decided to subset the review into focused subanalyses by creating a phased 2-step extraction approach. The first step extracted review characteristics (eg, bibliometric data, DHT type, health care setting) that were used to create subsets of smaller samples from the parent review. The second-step protocol was used to extract qualitative data on determinants of DHT implementation for the subsample focused on chronic disease prevention and management identified in step 1. Extraction protocols for both steps were iteratively developed and pilot tested to ensure clarity and consensus on data extraction.

The second data extraction protocol, which was used to extract qualitative data (text from review articles) on the barriers and facilitators of DHT implementation, was developed based on the Consolidated Framework for Implementation Research (CFIR), which comprehensively outlines implementation determinants across multiple levels: innovation (eg, complexity, design), implementer (eg, health care provider motivation and capability), inner setting (eg, communications, available resources), and outer setting (eg, policies and law, partnerships) (21). Reviewers extracted verbatim text from the included reviews that described determinants of DHT implementation and categorized the text to CFIR domains and constructs. If the description of an implementation determinant did not fit within an existing CFIR construct, reviewers placed the text into “other” categories in the appropriate CFIR level. Reviewers extracted each determinant as a barrier or facilitator if clearly indicated as such in the review. Reviewers categorized a determinant as both a barrier and a facilitator if the determinant was described as such by a review or if the language describing the determinant was neutral. Data were also extracted on the use of a theory, model, or framework (TMF) (yes or no), the name of the TMF, and how it was applied (eg, inform research questions or aims, support data extraction or analysis, interpret results, or frame discussion). Dual-blinded independent extraction and consensus meetings were used to ensure the reliability of data (A.S.G., A.E.G., L.P., M.K., D.T., O.T.N., K.T., W.T., B.W.). Reviewers abstracted data by copying and pasting text from each review into the appropriate CFIR construct in a Microsoft Excel (Microsoft Corporation) workbook. Following a consensus meeting, the final data were entered into a form in Qualtrics (Qualtrics, LLC).

### Data synthesis

Data were downloaded (A.S.G., A.E.G., M.M.K., L.P.) for 2 study team members to review and summarize results by creating themes of barriers and facilitators in each CFIR construct. Study team members independently reviewed the “other” category for emergent themes and met to discuss findings. The number of reviews providing data for each CFIR construct was calculated. Results were organized by CFIR domain or level by summarizing barriers and facilitators across the constructs using narrative synthesis.

Quality of reviews was assessed using the Joanna Briggs Institute critical appraisal checklist for systematic reviews and research synthesis through dual-blinded independent assessment and consensus meetings (A.S.G., A.E.G., L.P., M.K.) (22). The checklist consists of 11 questions designed to determine the quality and reliability of systematic reviews. To assess how many primary studies show up in multiple reviews for this chronic disease subanalysis review, the GROOVE (Graphical Representation of Overlap for OVErviews) tool was used to calculate the corrected covered area (how much the same primary studies are repeatedly represented across reviews) (23).

## Results

The final sample reviews of DHTs used for chronic disease prevention and management included 15 reviews published from 2017 through 2025; the articles were published from 1999 through 2025 (Table 2). Most were systematic reviews (n = 9) (4,24,26,27,29,31,33,34,36), followed by scoping reviews (n = 4) (25,30,32,35), 1 rapid review (28), and 1 narrative review (37). Nine reviews focused on only 1 DHT (4,24,26,28,31,33–36). The most common DHT was telemedicine (n = 10) (24–27,29,30,32,33,35,37). The rest of the reviews focused on EHRs (n = 5) (4,25,29,30,36), clinical decision support tools (n = 3) (25,30,31), artificial intelligence (AI) (n = 2) (28,34), health monitoring systems and wearable devices (n = 3) (25,29,30), and smartphone applications, text messaging, or telephone calls (n = 4) (27,30,32,37). No reviews specified a geographic context. Three reviews focused on outpatient routine care (33,34,36), 2 reviews focused on inpatient and outpatient routine care (4,27), and 1 review focused on inpatient settings (28). Six reviews applied 1 or multiple TMFs (4,26,29,31,34,37). Two of these reviews used TMFs to inform the research question (31,37), and all 6 reviews used the TMFs to support data analysis and interpret findings (4,26,29,31,34,37). Five reviews had a quality score of 100% (4,27,29,33,34), 9 had a score of 80% or higher (4,25,27,29–31,33,34,37), and no review had a score below 50%. The corrected covered area for the included reviews indicated slight overlap at 0.07%. Of the 388 parent studies, 384 were included in 1 review, and the remaining 4 were in 2 reviews.

**Table 2:** Characteristics of Studies Included (N = 15), Subanalysis of an Umbrella Review of Determinants of Digital Health Tool Implementation in Chronic Disease Prevention and Management in Clinical Practice Settings, 2013–2025

Lead author	Title	Dates of articles	No. of articles	Type of review	Type of digital health tool	Geographic context	Health care setting	Theory, model, or framework
Finnane et al (24), 2017	Teledermatology for the diagnosis and management of skin cancer: a systematic review	2010–2015	21	Systematic review	Telemedicine	Not specified	Not specified	—
Palacholla et al (25), 2019	Provider- and patient-related barriers to and facilitators of digital health technology adoption for hypertension management: scoping review	2008–2017	36	Scoping review	EHR; clinical decision support tool/system; telemedicine; health monitoring systems	Not specified	Not specified	—
Helleman et al (26), 2020	The current use of telehealth in ALS care and the barriers to and facilitators of implementation: a systematic review	2006–2019	16	Systematic review	Telemedicine	Not specified	Not specified	CFIR^a^
Foong et al (27), 2020	Facilitators and barriers of using digital technology for the management of diabetic foot ulcers: a qualitative systematic review	2008–2018	8	Systematic review	Telemedicine; smartphone application; website; text messaging	Not specified	Inpatient settings; outpatient routine care	—
Clement and Maldonado (28), 2021	Augmenting the transplant team with artificial intelligence: toward meaningful AI use in solid organ transplant	2020–2021	74	Rapid review	AI	Not specified	Inpatient settings	—
Hopstaken et al (29), 2021	Effect of digital care platforms on quality of care for oncological patients and barriers and facilitators for their implementation: systematic review	2000–2020	17	Systematic review	EHR; telemedicine; health monitoring systems	Not specified	Not specified	Grol and Wensing Implementation Framework^b^
Whitelaw et al (30), 2021	Barriers and facilitators of the uptake of digital health technology in cardiovascular care: a systematic scoping review	2008–2019	29	Scoping review	EHR; clinical decision support tool/system; Telemedicine; Smartphone application; wearable device; health monitoring systems	Not specified	Not specified	—
Sung et al (4), 2022	Using an integrated framework to investigate the facilitators and barriers of health information technology implementation in noncommunicable disease management: systematic review	xxxx–2022	51	Systematic review	EHR	Not specified	Inpatient settings; outpatient routine care	Consolidated Framework for Implementation Research (CFIR)^a^; Levels of Change Framework^c^
Bamgboje-Ayodele et al (31), 2023	Clinical decision support systems for chronic obstructive pulmonary disease (COPD) in hospitals: a systematic review	2014–2019	12	Systematic review	Clinical decision support tool/system	Not specified	Not specified	Reach, Effectiveness, Adoption, Implementation, and Maintenance Framework (RE-AIM)^d^
Ramachandran et al (32), 2023	Barriers and facilitators to the adoption of digital health interventions for COPD management: a scoping review	2008–2021	27	Scoping review	Telemedicine; text messaging; telephone support	Not specified	Not specified	—
Rosenstrom et al (33), 2024	Nurses’ experiences with virtual consultations and home-monitoring in patients with cardiac disease: a systematic review and qualitative meta-synthesis of results	2014–2022	10	Systematic review	Telemedicine	Not specified	Outpatient routine care	—
Goh et al (34), 2025	Challenges in implementing artificial intelligence in breast cancer screening programs: systematic review and framework for safe adoption	2019–2023	20	Systematic review	AI	Not specified	Outpatient routine care	CFIR^a^; AI Governance framework^e^
Kissi and Annobil (35), 2025	The advantageous impact of telestroke: global insights and implications for Africa: a scoping review of literature	2017–2023	31	Scoping review	Telemedicine	Not specified	Not specified	—
Naous et al (36), 2025	A systematic review on the impact of electronic consultations on diabetes care	2009–2023	6	Systematic review	EHR	Not specified	Outpatient routine care	—
Salmi et al (37), 2025	Facilitators and barriers affecting the implementation of e-health for chronic respiratory diseases in remote settings: a qualitative evidence synthesis	1999–2023	41	Narrative review	Telemedicine; smartphone application; text messaging; telephone support	Not specified	Outpatient routine care	The RETREAT framework^f^ DHEF^g^

Abbreviations: —, not applicable; AI, artificial intelligence; CFIR, Consolidated Framework for Implementation Research; DHEF, Digital Health Equity Framework; EHR, electronic health record.

a Damschroder et al (21).

b Grol and Wensing (38).

c Ferlie and Shortell (39).

d Glasgow et al (40).

e Goh et al (34).

f Booth et al (41).

g Richardson et al (42).

### Innovation determinants

Among the reviews, the most frequently identified innovation-level determinants included evidence base (barriers, n = 8 reviews [4,25,27–30,33,34]; facilitators, n = 2 reviews [4,37]), relative advantage (barriers, n = 12 reviews [4,24–30,32–34,37]; facilitators, n = 9 reviews [4,25–27,29,30,32,35,37]), and design (barriers, n = 10 reviews [4,24,26,27,29,30,32,34,35,37]; facilitators, n = 5 reviews [4,24,26,27,37]) (Table 3). The evidence base for DHTs in chronic disease prevention and management more often acted as a barrier than a facilitator, with many reviews indicating a lack of evidence for the positive clinical impact of using DHTs. The concern about clinical impact was prominent for AI tools but described as a barrier across DHTs, with questions raised around the clinical, social, and economic benefits of using these tools in clinical care settings. Relative advantage was described as both a barrier and facilitator to DHT implementation. Many reviews highlighted how DHTs were better than current practice (ie, improvements in the quality, efficiency, and cost of care). DHTs were noted for improving diagnoses, monitoring disease progress, facilitating disease management, increasing provider–patient communication, and increasing access to chronic disease specialists and care. However, DHTs were also seen as less advantageous than traditional care, mostly due to the perception that DHTs dehumanized interactions between providers and patients and may provide unreliable, insufficient, or invalid data for clinical decisions and care, leading to worse health outcomes. Design was identified as a critical component to DHT implementation. DHTs that were not well designed, did not consider clinical workflows, did not include clinician input in design, had low functionality in practice, or had technical issues were less likely to be adopted and used by providers. However, well-designed DHTs that were easy to use and built into clinical workflows were more likely to be adopted and used by providers.

**Table 3:** CFIR Constructs^a^ Described as Determinants, Subanalysis (N = 15 Studies) of an Umbrella Review of Determinants of Digital Health Tool Implementation in Chronic Disease Prevention and Management in Clinical Practice Settings, 2013–2025

CFIR construct	Barrier: no. of reviews	Barrier: quote	Facilitator: no. of reviews	Facilitator: quote
**Innovation**				
Evidence base	8	Although there is a growing body of literature that describes the positive effects of digital care platforms, high-quality studies describing the effectiveness of these platforms integrated in oncological care are currently lacking. Hopstaken et al (29)	2	Evidence of the efficacy of e-health for improving care was seen as important for both patients as well as for HCPs: “I think that type of evidence is what’s going to change my mind as a practitioner about whether it’s worth using it or not.” Salmi et al (37)
Relative advantage	12	The use of asynchronous digital technology reduces patient engagement. Patients feel less responsible and empowered in handling their conditions because their care is discussed by the visiting nurse and the hospital staff without patients' direct involvement. . . . Both physicians and nurses reported that face-to-face consultation with the patient was important in medical consultation. Some felt that telemedicine reduced direct contact in care provision and might lead patients to complain about not seeing a physician. One doctor felt reluctant to switching to solely computer-based consultation in the future. Foong et al (27)	9	Neurologists acknowledged the significant enhancement of work-life balance through reduced travel time, effectively bridging the gap between patients’ local care needs and physicians’ need for improved equilibrium. Kissi and Annobil (35)
Adaptability	7	Lack of customizable features. Whitelaw et al (30)	4	Technology enables individualized care. Foong et al (27)
Complexity	4	Data-related problems (collecting, managing, processing), technical challenges, and overall complexity. Sung et al (4)	4	Perceived ease of use. Ramachandran et al (32)
Design	10	In terms of the AI user interface, through qualitative interviews, Hendrix et al found that 26%–33% of radiologists were deterred if the AI features did not align with their preferences, highlighting the need for increased collaboration between radiologists and technical professionals to bridge this gap. Goh et al (34)	5	Good data quality, good design, good data visualization, good content (eg, specific), and ease of use. Sung et al (4)
Cost	4	Uncertainty about costs caused scepticism and reluctance to engage in e-health. Salmi et al (37)	3	For HCPs, the use of e-health was facilitated if the intervention was perceived as cost-effective for the health care facilities they were working in. Helleman et al (26)
**Implementer**				
Capability	6	Lack of knowledge . . . lack of digital skills. Rosenstrom et al (33)	0	NA
Opportunity	8	HCPs indicated that they have a lack of time, they did not have enough time to adjust, or the use of HIT increased consultation time and therefore depleted time resources. HCPs often had competing priorities in work and perceived the newly implemented HIT as noncore work activity. Sung et al (4)	0	NA
Motivation	6	Some nurses reported working in a constantly changing work environment, which could decrease their motivation for implementing new workflows offered by new technology. Rosenstrom et al (33)	4	HCP interest in learning new technologies. Salmi et al (37)
Need	1	Sometimes physicians preferred traditional health care message and thought that change is unneeded, especially because they did not believe patients could not efficiently manage their data. Sung et al (4)	2	Patient–technology–provider relationship: e-health significantly impacted patient–HCP relationships and communication patterns, with reduced face-to-face interaction emerging as a key sub-theme. This allowed HCPs to manage more patients, decreased the need for physical infrastructure, and, during the COVID-19 pandemic, reduced the risk of transmission. Salmi et al (37)
**Inner setting**				
Structural characteristics	7	Lack of leadership and organizational support. Palacholla et al (25)	6	Approval and organizational support from cardiology departments and/or hospitals. Whitelaw et al (30)
Culture	0	NA	2	Innovation-oriented culture. Sung et al (4)
Compatibility	7	Two studies mentioned the integration of the platform into existing systems as a barrier. This would be especially relevant in cases in which the digital care platforms were also developed for other chronic diseases. Hopstaken et al (29)	5	Alignment of DHI usage to current practice and policy. Ramachandran et al (32)
Incentive systems	3	Reimbursement issues. Whitelaw et al (30)	1	Internal factors encompass payer mix, patient volume, and hospital profitability. Kissi and Annobil (35)
Available resources	7	Technological prerequisites such as comprehensive network coverage, stable internet connectivity, and affordable data services are critical for effective telestroke implementation. Kissi and Annobil (35)	5	Tech support. Palacholla et al (25)
Access to knowledge and information	7	The lack of clear guidelines discourages health care providers from integrating telestroke into routine clinical practice. Kissi and Annobil (35)	7	While e-health had the potential to positively impact health-seeking behavior, the benefits could be hampered by problems with training. Adequate training for both patients and HCPs emerged as a key factor to enable technology access and the implementation of e-health interventions. Salmi et al (37)
**Outer setting**				
Critical incidents	0	NA	1	The COVID-19 pandemic proved to be an effective facilitator for the use of e-health. Some studies cited that because of the pandemic, the number of clinic visits was reduced and replaced by e-health. This was attributed to the desire to decrease contagion, as well as the belief that e-health was more accessible than in-person consults. Salmi et al (37)
Local attitudes	1	A cultural belief referred to as “Appalachian pride” could be a barrier for telehealth adoption. Appalachian pride comprises multiple cultural values (eg, privacy) that are upheld in tight-knit communities. Appalachian pride may impact tele-health adoption and preferences [US, North Carolina]. Salmi et al (37)	0	—
Local conditions	2	The availability of a reliable internet or phone connection represented an important enabler to the use of e-health. Local network issues resulting in poor audio connection, or “freezing” of the app and upload issues led to underuse and delays in patient care. Some studies noted that the greatest trigger for introducing e-health was the absence of pre-established infrastructure or services. Examples included places without access to basic health resources, and locations where specialist services were unavailable. Salmi et al (37)	3	Key external factors include market competition and critical access hospital status. Kissi and Annobil (35)
Partnerships and connections	4	A lack of coordination between vendors and the hospital, nurses, and providers challenged the implementation process. Sung et al (4)	3	A coordinated effort among governments, health care institutions, private sector stakeholders, and international organizations may be essential in creating a resilient and scalable telestroke ecosystem across the continent. Kissi and Annobil (35)
Policies and laws	5	The absence of clear regulations can create uncertainty for stakeholders about the legal boundaries associated with AI adoption. Goh et al (34)	2	Policymakers may prioritize the development of standardized regulatory frameworks that provide clear guidelines on ethical, legal, and operational aspects of telestroke, ensuring compliance with data security, patient confidentiality, and informed consent requirements. Kissi and Annobil (35)
Funding	0	NA	1	Sustainable financing mechanisms, including the integration of telestroke into national health care budgets and the promotion of public–private collaborations, may reduce dependency on external funding and ensure long-term viability. Kissi and Annobil (35)

Abbreviations: AI, artificial intelligence; CFIR, Consolidated Framework for Implementation Research; DHI, digital health information; HCPs, health care providers; HIT, health information technology; NA, not applicable.

a Certain constructs within each domain did not show up in any reviews: innovation: source, trialability; inner setting: relational connections, communications, tension for change, relative priority, mission alignment; outer setting: external pressure.

Less frequently cited determinants were adaptability (barriers, n = 7 reviews [4,25,28,30,32,33,37]; facilitators, n = 4 reviews [4,25,27,37]), complexity (barriers, n = 4 reviews [4,25,26,37]; facilitators, n = 4 reviews [25,26,32,37]), and cost (barriers, n = 4 reviews [4,27,30,37]; facilitators, n = 3 reviews [4,26,37]). Adaptable tools that were customizable to different settings, populations, languages, or diseases were seen as easier to adopt and implement. Complexity acted as both a facilitator and barrier to implementation. DHTs that were slow and cumbersome and complicated to use (eg, generating output that was hard to interpret), were harder to implement. Conversely, DHTs with clear, simple language and easy-to-interpret output were easier to adopt and use. Cost appeared across a few reviews; the perceived or actual DHT cost-effectiveness influenced the likelihood of implementation.

### Implementer (health care provider) determinants

Implementer opportunity (barriers, n = 8 reviews [4,25,26,29,30,32,33,37]; facilitators, n = 0 reviews) and capability (barriers, n = 6 reviews [4,27,32,33,35,36]; facilitators, n = 0 reviews) were described by reviews as barriers to DHT implementation (Table 3). Reviews described an overall lack of opportunities to adopt and use DHTs for chronic disease prevention and management. Health care providers were described as having limited time and resources for DHT training. In the early stages of adoption, using the DHT was perceived as more time consuming, which may have implications for continued use. Implementing DHTs was described as additional effort amid competing work priorities. Provider capability, overall lack of digital skills, familiarity, knowledge, and apprehension about something new were barriers in DHT implementation.

Other implementer determinants described in the reviews were motivation (barriers, n = 6 reviews [4,27,33,34,36,37]; facilitators, n = 4 reviews [4,27,30,37]) and need (barriers, n = 1 review [4]; facilitators, n = 2 reviews [27,37]). Providers were described as lacking motivation to implement DHTs due to already managing a constantly changing work environment, wanting to maintain their usual way of practice, and lacking interest in DHTs. However, reviews described motivation as a facilitator among providers who were eager to learn new technologies and had positive attitudes about DHTs. Need was described in only a few reviews but highlighted the tension between whether DHTs were perceived as needed in clinical care. For example, during the height of the COVID-19 pandemic, telemedicine addressed implementers’ need for maintaining continuity of chronic disease management and care while minimizing their own risk of infection.

### Inner setting (clinical setting) determinants

The most prominent inner setting determinants included access to knowledge and information (barriers, n = 7 reviews [4,27,32–35,37]; facilitators, n = 7 reviews [26,27,30,32,33,35,37]), structural characteristics (barriers, n = 7 reviews [4,25,27,29,32,33,37]; facilitators, n = 6 reviews [4,25,27,29,30,33]), available resources (barriers, n = 7 reviews [4,26,27,32,33,35,37]; facilitators, n = 5 reviews [4,25,30,35,37]), and compatibility (barriers, n = 7 reviews [4,25,27,29,32,33,37]; facilitators, n = 5 reviews [4,24,25,29,32]) **(** Table 3 **)**. Access to knowledge and information (eg, lack of training in DHT use, limited guidelines and protocols to ensure patient safety and provider confidence) was described as a key barrier to DHT implementation. Training in using DHTs (eg, technical aspects, clinical care application) and providing ongoing technical support were described as facilitators to DHT implementation. Structural characteristics, such as leadership and organizational support, administrative support, and information technology (IT) infrastructure, and available resources, such as funding, available workforce, internet connectivity, and time, influenced the implementation of DHTs. Some reviews described how structural characteristics and available resources were more likely to act as barriers in rural areas and in low- and middle-income countries, where resources and infrastructure are more limited. Compatibility and how well a DHT was integrated into existing systems, including clinical workflow, existing electronic platforms, and IT infrastructure, was described by reviews as both a barrier and facilitator to DHT implementation.

Culture (barriers, n = 0 reviews; facilitators, n = 2 reviews [4,37]), and incentive systems (barriers, n = 3 reviews [30,35,37]; facilitators, n = 1 review [35]) were less commonly discussed determinants. Two reviews described how the culture of a health care system or hospital that was open to innovation and change could facilitate the adoption and implementation of DHTs. Some reviews noted that incentive systems, such as insurance reimbursement and financial remuneration for health care providers for using DHTs, could facilitate DHT implementation.

### Outer setting (clinical care external environment) determinants

Outer setting determinants that came up in multiple reviews included policies and laws (barriers, n = 5 reviews [4,29,34,35,37]; facilitators, n = 2 reviews [35,37]), partnerships and connections (barriers, n = 4 reviews [4,29,33,35]; facilitators, n = 3 reviews [33,35,37]), and local conditions (barriers, n = 2 reviews [35,37]; facilitators, n = 3 reviews [29,35,37]) (Table 3). Policies and laws were described as both a barrier to and a facilitator of DHT implementation. Reviews indicated the need for regulation of DHTs to ensure data security and privacy, reduce ambiguity around liability related to AI-enabled DHT errors leading to patient harm or misdiagnosis, and uphold oversight of DHT development and use. Reviews underscored that implementation requires robust legal and governance frameworks that are currently limited and insufficiently established. Reviews discussed the need for partnerships and connections to facilitate implementation. Throughout all phases of implementation, from development and testing to implementation and sustainability, reviews noted the importance of collaborations among digital tool developers, health care providers, health systems, government, and communities to ensure acceptability, feasibility, and sustainability of DHTs in practice. Local conditions were also described as being influential for DHT implementation. Reviews noted that internet connectivity, market competition, and health care availability may influence the adoption and implementation of DHTs.

Funding (barriers, n = 0 reviews; facilitators, n = 1 review [35]), local attitudes (barriers, n = 1 review [37]; facilitators, n = 0 reviews), and critical incidents (barriers, n = 0 reviews; facilitators, n = 1 reviews [37]) were less commonly discussed in the included reviews. Securing long-term and reliable funding was described in 1 review as important for sustainability of DHTs. Local attitudes were described as barriers in which cultural context and values may not align with use of DHTs (eg, values and comfort related to privacy and human relationships). DHTs that are adaptable to local context were described as more likely to overcome these barriers. Critical incidents were only described as facilitators of DHT implementation, such as the role of the COVID-19 pandemic in catalyzing the adoption and use of DHTs for chronic disease prevention and management.

## Discussion

We found that implementation of DHTs for chronic disease prevention and management is shaped by multilevel determinants across CFIR domains. Most determinants identified across CFIR levels were barriers, and when facilitators were described, many reflected the inverse of those barriers. The most salient determinants for implementing DHTs were workflow integration, adaptability, provider education and support, and infrastructure. These findings reflect implementation determinants of DHTs for other contexts (eg, mental health, rehabilitation, pediatrics), such as fit with workflow, supportive infrastructure including EHR integration, and organizational policies to incentivize adoption and use (43,44). 

At the innovation level, workflow and adaptability emerged as both barriers and facilitators, depending on how well DHTs were aligned with existing clinical processes and could be tailored to specific diseases and populations. For chronic disease care, digital tools must fit into workflows in ways that reduce burden on providers while supporting care coordination across primary care, specialty care, nursing, pharmacy, and other team members. This finding is especially important for chronic conditions that require sustained management, frequent follow-up, titration of medication, and communication across settings (45). DHTs that support ongoing monitoring of symptoms, blood pressure, glucose, medication adherence, and other disease-specific indicators may improve care only when they are integrated into clinical workflows that allow timely review, response, and escalation when needed (6,16).

The adaptability of DHTs is also critical for chronic disease management because patient needs often change over time and care typically requires multidisciplinary coordination and proactive monitoring (45). Tools that can be tailored to different disease trajectories, patient preferences, and care team roles may be more feasible to implement and sustain. Adaptability may be particularly important for promoting equity in settings that serve patients with limited access to broadband, smartphones, or stable internet connectivity, as well as for patients with limited digital literacy or health literacy (2,46). If DHTs are not designed with these realities in mind, they may unintentionally widen disparities in access to chronic disease prevention and management services.

At the implementer level, providers in clinical settings need adequate training and ongoing support to use digital tools effectively within routine chronic disease care. Training should include guidance on how to interpret patient-generated data, communicate findings to patients, and incorporate digital information into shared decision-making. Providers also need evidence that DHTs improve outcomes and do not disrupt the therapeutic relationship (6,9–11). Maintaining interpersonal connection is particularly important in chronic disease care, where trust and continuity are central to helping patients self-monitor, make behavioral changes, and adhere to treatment plans (47).

At the inner and outer setting levels, implementation depends on organizational and policy support. Clinics need personnel, protocols, and technical infrastructure to support DHT use, including integration with the EHR, processes for reviewing remote monitoring data, and clear roles for responding to alerts or abnormal readings. Because chronic disease management often involves repeated data collection over time, sustained coordination among providers, communication with patients between visits, strong infrastructure, and IT support are essential for effective implementation and sustained use (14,48). Without these supports, digital tools may create additional workload rather than improving care delivery.

### Implications for practice and research

Results of this study can be used to inform implementation strategies for DHTs in chronic disease prevention and management in clinical settings. We used the updated CFIR framework, a common and expansive tool that comprehensively assesses several levels, to classify determinants. Because our study considered determinants across contexts and digital tool types, clinics and providers can use these findings for preemptive strategies to reduce ineffective implementation and promote sustained use. The CFIR-ERIC (Expert Recommendations for Implementing Change) implementation strategy matching tool could be a useful process that clinics and providers can use to select and implement strategies to address barriers (49). Its use coincides with the methods of this review, as the barriers identified are categorized appropriately by CFIR level.

### Limitations and strengths

Our team followed rigorous and appropriate procedures and used an implementation science framework for understanding determinants. Although this study allowed us to comprehensively understand determinants across contexts and DHT types, it has several limitations. Generalizability is constrained by incomplete reporting, limited variability in setting and type of DHT, and the small sample size. The limited sample size and variation in how included reviews reported results also restricted our ability to examine differences in functionality, complexity, use, and implementation determinants by DHT type and across individual chronic diseases and geographic contexts. A larger sample size and less variation could have yielded a more nuanced and broadly applicable synthesis. Additionally, this subanalysis was limited to reviews included in the parent review. It is possible that other eligible reviews were not identified.

Although classifying determinants using CFIR was a strength of our review, this approach may have omitted levels and domains that may have been identified had another TMF been used to guide synthesis of our results. Although CFIR is commonly used and is a comprehensive determinants framework, it may have reduced applicability outside the US (21). Because reviews did not report geographic location, however, we do not know whether this factor may have influenced our results.

### Conclusion

This was the first study to understand determinants of DHT implementation to address chronic disease prevention and management in clinical settings. Our novel approach and rigorous methods will aid researchers, health care administrators, and policy makers in understanding and addressing determinants to improve the adoption and sustainability of effective DHTs for people with chronic disease.

## Appendix Search Strategy for an Umbrella Review of Determinants of Digital Health Tool Implementation in Chronic Disease Prevention and Management in Clinical Practice Settings, 2013–2025

**PubMed (filters: systematic review; publication date 2013–2025)**

Computers, Handheld"[Mesh] OR "User-Computer Interface"[Mesh] OR "Telephone"[Mesh:NoExp] OR "Cell Phone"[Mesh] OR "Artificial Intelligence"[Mesh:NoExp] OR "Machine Learning"[Mesh] OR "Telemedicine"[Mesh] OR "biomedical technology"[Mesh] OR "medical informatics"[Mesh] OR "remote sensing technology"[Mesh:NoExp] OR "electronic health records"[Mesh] OR "wearable electronic devices"[Mesh] OR "mobile applications"[Mesh] OR "handheld computer*"[tiab] OR "User-Computer Interfac*"[tiab] OR "telephone*"[tiab] OR “cell phone*”[tiab] OR "Artificial Intelligence"[tiab] OR "Machine Learning"[tiab] OR "Telemedicine"[tiab] OR "biomedical technolog*"[tiab] OR "medical informatic*"[tiab] OR "remote sensing technolog*"[tiab] OR "electronic health record*"[tiab] OR "wearable electronic device*"[tiab] OR "mobile applicat*"[tiab] OR "artificial intelligence"[tiab] OR "augmented reality"[tiab] OR "cellular phone*"[tiab] OR "clinical decision support"[tiab] "computerised medical record*"[tiab] OR "computerized medical record*"[tiab] OR "decision support system*"[tiab] OR "deep learning"[tiab] OR "digital health*"[tiab] OR "digital therapeutic*"[tiab] OR ehealth[tiab] OR "e health"[tiab] OR "electronic health record*"[tiab] OR "electronic medical record*"[tiab] OR EMR[tiab] OR "health application*"[tiab] OR "health app*"[tiab] OR "health informatic*"[tiab] OR "health information technology"[tiab] OR "health IT"[tiab] OR "health tech*"[tiab] OR "health wearable*"[tiab] OR "health care application*"[tiab] OR "health care apps"[tiab] OR "health care informatic*"[tiab] OR "health care information technology"[tiab] OR "health care IT"[tiab] OR "health care technolog*"[tiab] OR "healthcare apps"[tiab] OR "healthcare informatic*"[tiab] OR "healthcare information technology"[tiab] OR "healthcare IT"[tiab] OR "healthcare technolog*"[tiab] OR "healthcare wearable*"[tiab] OR "healthcare IoT"[tiab] OR healthtech[tiab] OR "internet of things"[tiab] OR "machine intelligence"[tiab] OR "machine learning"[tiab] OR "medical informatics"[tiab] OR mhealth[tiab] OR "m health"[tiab] OR "mobile application*"[tiab] OR "mobile app*"[tiab] OR "mobile device*"[tiab] OR "mobile health*"[tiab] OR "mobile phone*"[tiab] OR "mobile technolog*"[tiab] OR "remote monitoring"[tiab] OR "remote patient monitoring"[tiab] OR "smart phone*"[tiab] OR smartphone*[tiab] OR SMS[tiab] OR telecare[tiab] OR "tele care"[tiab] OR teleconsultation*[tiab] OR "tele consultation*"[tiab] OR telehealth[tiab] OR "tele health"[tiab] OR telemedicine[tiab] OR "tele medicine"[tiab] OR "text messag*"[tiab] OR "virtual care"[tiab] OR "virtual consultation*"[tiab] OR "virtual health care"[tiab] OR "virtual healthcare"[tiab] OR "virtual medicine"[tiab] OR "virtual systems"[tiab] OR "wearable device*"[tiab] OR "wearable electronic device*"[tiab] OR "wearable technolog*"[tiab] OR “health databases”[tiab] OR “health information technology”[tiab] OR “digital imaging”[tiab] OR “wearable sensor*”[tiab] OR “technology, medical”[tiab] OR “patient record system*”[tiab] OR “medical record*”[tiab] OR “health information management service”[tiab] OR “health information management”[tiab] OR “digital technology”[tiab] OR “health information network*”[tiab] OR "telephone triage nurs*"[tiab] AND "Diffusion of Innovation"[Mesh] OR "Diffusion of Innovation"[tiab] OR adopt*[tiab] OR advance*[tiab] barrier*[tiab] OR catalyst*[tiab] OR challenge*[tiab] OR constraint*[tiab] OR contributor*[tiab] OR deploy*[tiab] OR enabler*[tiab] OR enhance*[tiab] OR execut*[tiab] OR facilitat*[tiab] OR helper*[tiab] OR hindrance*[tiab] OR hurdle*[tiab] OR impediment*[tiab] OR implement*[tiab] OR integrat*[tiab] OR limitation*[tiab] OR pitfall*[tiab] OR promot*[tiab] OR restriction*[tiab] OR setback*[tiab] OR supporter*[tiab] OR "utilis*"[tiab] OR "utiliz*"[tiab] AND"Ambulatory Care Facilities"[tiab] OR "hospital outpatient clinic"[tiab] OR "Safety net Provider*"[tiab] OR "Academic Medical Center*"[tiab] OR "Community Health Worker*"[tiab] OR "Nurse Clinician*"[tiab] OR physician*[tiab] OR "Patient Care Team"[tiab] OR "Physician Assistant*"[tiab] OR nurse*[tiab] OR nutritionist*[tiab] OR "ambulatory care facilit*"[tiab] OR "hospital outpatient clinic*"[tiab] OR "outpatient clinic*"[tiab] OR "safety net provider*"[tiab] OR "academic medical cent*"[tiab] OR "community health worker*"[tiab] OR "nurse clinician*"[tiab] OR physician*[tiab] OR "patient care team"[tiab] OR "physician assistant*"[tiab] OR nurse*[tiab] OR nutritionist*[tiab] OR "care delivery setting*"[tiab] OR "care facilit*"[tiab] OR "child guidance clinic*"[tiab] OR "clinical care setting*"[tiab] OR "clinical environment*"[tiab] OR "clinical facilit*"[tiab] OR "clinical practice setting*"[tiab] OR "community health cent*"[tiab] OR "community mental health cent*"[tiab] OR "free standing clinic*"[tiab] OR "health care environment*"[tiab] OR "healthcare environment*"[tiab] OR "health care facilit*"[tiab] OR "healthcare facilit*"[tiab] OR "health care institution*"[tiab] OR "healthcare institution*"[tiab] OR "health care organisation*"[tiab] OR "healthcare organisation*"[tiab] OR "health care organization*"[tiab] OR "healthcare organization*"[tiab] OR "health care system*"[tiab] OR "healthcare system*"[tiab] OR hospital*[tiab] OR "hospital setting*"[tiab] OR "maternal child health cent*"[tiab] OR "medical clinic*"[tiab] OR "medical institution*"[tiab] OR "out patient clinic*"[tiab] OR "outpatient clinic*"[tiab] OR "pain clinic*"[tiab] OR "safety net clinic*"[tiab] OR "safety net hospital*"[tiab] OR surgicent*[tiab] OR "surgery cent*"[tiab] OR "teaching hospital*"[tiab] OR "treatment cent*"[tiab] OR "university hospital*"[tiab] OR "university medical cent*"[tiab] OR "urgent care cent*"[tiab] OR "urgent care clinic*"[tiab] OR "care delivery setting*"[tiab] OR "care facilit*"[tiab] OR "child guidance clinic*"[tiab] OR "mobile health units"[tiab]

**Scopus (filters: range 2013–2025; search within article title, abstract, keywords)**

"handheld computer*" OR "User-Computer Interfac*" OR "telephone*" OR “cell phone*” OR "Artificial Intelligence" OR "Machine Learning" OR "Telemedicine" OR "biomedical technolog*" OR "medical informatic*" OR "remote sensing technolog*" OR "electronic health record*" OR "wearable electronic device*" OR "mobile applicat*" OR "artificial intelligence" OR "augmented reality" OR "cellular phone*" OR "clinical decision support" "computerised medical record*" OR "computerized medical record*" OR "decision support system*" OR "deep learning" OR "digital health*" OR "digital therapeutic*" OR ehealth OR "e health" OR "electronic health record*" OR "electronic medical record*" OR EMR OR "health application*" OR "health app*" OR "health informatic*" OR "health information technology" OR "health IT" OR "health tech*" OR "health wearable*" OR "health care application*" OR "health care apps" OR "health care informatic*" OR "health care information technology" OR "health care IT" OR "health care technolog*" OR "healthcare apps" OR "healthcare informatic*" OR "healthcare information technology" OR "healthcare IT" OR "healthcare technolog*" OR "healthcare wearable*" OR "healthcare IoT" OR healthtech OR "internet of things" OR "machine intelligence" OR "machine learning" OR "medical informatics" OR mhealth OR "m health" OR "mobile application*" OR "mobile app*" OR "mobile device*" OR "mobile health*" OR "mobile phone*" OR "mobile technolog*" OR "remote monitoring" OR "remote patient monitoring" OR "smart phone*" OR smartphone* OR SMS OR telecare OR "tele care" OR teleconsultation* OR "tele consultation*" OR telehealth OR "tele health" OR telemedicine OR "tele medicine" OR "text messag*" OR "virtual care" OR "virtual consultation*" OR "virtual health care" OR "virtual healthcare" OR "virtual medicine" OR "virtual systems" OR "wearable device*" OR "wearable electronic device*" OR "wearable technolog*" OR “health databases” OR “health information technology” OR “digital imaging” OR “wearable sensor*” OR “technology, medical” OR “patient record system*” OR “medical record*” OR “health information management service” OR “health information management” OR “digital technology” OR “health information network*” OR "telephone triage nurs*" AND “Diffusion of Innovation" OR adopt* OR advance* barrier* OR catalyst* OR challenge* OR constraint* OR contributor* OR deploy* OR enabler* OR enhance* OR execut* OR facilitat* OR helper* OR hindrance* OR hurdle* OR impediment* OR implement* OR integrat* OR limitation* OR pitfall* OR promot* OR restriction* OR setback* OR supporter* OR "utilis*" OR "utiliz*" AND "Ambulatory Care Facilities" OR "hospital outpatient clinic" OR "Safety net Provider*" OR "Academic Medical Center*" OR "Community Health Worker*" OR "Nurse Clinician*" OR physician* OR "Patient Care Team" OR "Physician Assistant*" OR nurse* OR nutritionist* OR "ambulatory care facilit*" OR "hospital outpatient clinic*" OR "outpatient clinic*" OR "safety net provider*" OR "academic medical cent*" OR "community health worker*" OR "nurse clinician*" OR physician* OR "patient care team" OR "physician assistant*" OR nurse* OR nutritionist* OR "care delivery setting*" OR "care facilit*" OR "child guidance clinic*" OR "clinical care setting*" OR "clinical environment*" OR "clinical facilit*" OR "clinical practice setting*" OR "community health cent*" OR "community mental health cent*" OR "free standing clinic*" OR "health care environment*" OR "healthcare environment*" OR "health care facilit*" OR "healthcare facilit*" OR "health care institution*" OR "healthcare institution*" OR "health care organisation*" OR "healthcare organisation*" OR "health care organization*" OR "healthcare organization*" OR "health care system*" OR "healthcare system*" OR hospital* OR "hospital setting*" OR "maternal child health cent*" OR "medical clinic*" OR "medical institution*" OR "out patient clinic*" OR "outpatient clinic*" OR "pain clinic*" OR "safety net clinic*" OR "safety net hospital*" OR surgicent* OR "surgery cent*" OR "teaching hospital*" OR "treatment cent*" OR "university hospital*" OR "university medical cent*" OR "urgent care cent*" OR "urgent care clinic*" OR "care delivery setting*" OR "care facilit*" OR "child guidance clinic*" OR "mobile health units" AND Systematic AND Review

**CINAHL (filters: published date, January 2013–June 2025, publication type: systematic review)**

(MH "Mobile Health Units") OR (MH "Telehealth") OR (MH "Cellular Phone") OR (MH "Mobile Applications") OR (MH "Health Information Networks") OR (MH "Health Information Systems") OR (MH "Databases, Health") OR (MH "Electronic Health Records") OR (MH "Patient Record Systems") OR (MH "Medical Records") OR (MH "Health Information Management Service") OR (MH "Health Information Management") OR (MH "Technology, Medical") OR (MH "Medical Informatics") OR (MH "Wearable Sensors") OR (MH "Artificial Intelligence") OR (MH "Digital Health") OR (MH "Digital Technology") OR (MH "Digital Imaging") OR (MH "Computers, Hand-Held") OR (MH "Telephone Triage Nursing") AND MH (MH "Diffusion of Innovation") OR TI ( "Diffusion of Innovation" OR adopt* OR advance* barrier* OR catalyst* OR challenge* OR constraint* OR contributor* OR deploy* OR enabler* OR enhance* OR execut* OR facilitat* OR helper* OR hindrance* OR hurdle* OR impediment* OR implement* OR integrat* OR limitation* OR pitfall* OR promot* OR restriction* OR setback* OR supporter* OR "utilis*" OR "utiliz*" ) OR AB ( "Diffusion of Innovation" OR adopt* OR advance* barrier* OR catalyst* OR challenge* OR constraint* OR contributor* OR deploy* OR enabler* OR enhance* OR execut* OR facilitat* OR helper* OR hindrance* OR hurdle* OR impediment* OR implement* OR integrat* OR limitation* OR pitfall* OR promot* OR restriction* OR setback* OR supporter* OR "utilis*" OR "utiliz*" ) AND MH ( (MH "Ambulatory Care Facilities") OR (MH "Surgicenters") OR (MH "Ambulatory Care") ) OR TI ( "Ambulatory Care Facilities" OR "hospital outpatient clinic" OR "Safety net Provider*" OR "Academic Medical Center *" OR "Community Health Worker*” OR "Nurse Clinician*” OR "Physicians " OR "Patient Care Team" OR "Physician Assistant*” OR "Nurse*” OR "Nutritionist*” OR "ambulatory care facilit*" OR "hospital outpatient clinic*" OR "outpatient clinic*" OR "safety net provider*" OR "academic medical cent*" OR "community health worker*" OR "nurse clinician*" OR "physician*" OR "patient care team" OR "physician assistant*" OR "nurse*" OR "nutritionist*" OR "care delivery setting*" OR "care facilit*" OR "child guidance clinic*" OR "clinical care setting*" OR "clinical environment*" OR "clinical facilit*" OR "clinical practice setting*" OR "community health cent*" OR "community mental health cent*" OR "free standing clinic*" OR "health care environment*" OR "healthcare environment*" OR "health care facilit*" OR "healthcare facilit*" OR "health care institution*" OR "healthcare institution*" OR "health care organisation*" "healthcare organisation*" OR "health care organization*" OR "healthcare organization*" OR "health care system*" OR "healthcare system*" OR hospital* OR "hospital setting*" OR "maternal child health cent*" OR "medical clinic*" OR "medical institution*" OR "out patient clinic*" OR "outpatient clinic*" OR "pain clinic*" OR “safety net clinic*” OR “safety net hospital*” OR surgicent* OR “surgery cent*” OR "teaching hospital*" OR "treatment cent*" OR "university hospital*" OR "university medical cent*" OR "urgent care cent*" OR "urgent care clinic*" OR "care delivery setting*" OR "care facilit*" OR "child guidance clinic*" ) OR AB ( "Ambulatory Care Facilities" OR "hospital outpatient clinic" OR "Safety net Provider*" OR "Academic Medical Center *" OR "Community Health Worker*” OR "Nurse Clinician*” OR "Physicians " OR "Patient Care Team" OR "Physician Assistant*” OR "Nurse*” OR "Nutritionist*” OR "ambulatory care facilit*" OR "hospital outpatient clinic*" OR "outpatient clinic*" OR "safety net provider*" OR "academic medical cent*" OR "community health worker*" OR "nurse clinician*" OR "physician*" OR "patient care team" OR "physician assistant*" OR "nurse*" OR "nutritionist*" OR "care delivery setting*" OR "care facilit*" OR "child guidance clinic*" OR "clinical care setting*" OR "clinical environment*" OR "clinical facilit*" OR "clinical practice setting*" OR "community health cent*" OR "community mental health cent*" OR "free standing clinic*" OR "health care environment*" OR "healthcare environment*" OR "health care facilit*" OR "healthcare facilit*" OR "health care institution*" OR "healthcare institution*" OR "health care organisation*" "healthcare organisation*" OR "health care organization*" OR "healthcare organization*" OR "health care system*" OR "healthcare system*" OR hospital* OR "hospital setting*" OR "maternal child health cent*" OR "medical clinic*" OR "medical institution*" OR "out patient clinic*" OR "outpatient clinic*" OR "pain clinic*" OR “safety net clinic*” OR “safety net hospital*” OR surgicent* OR “surgery cent*” OR "teaching hospital*" OR "treatment cent*" OR "university hospital*" OR "university medical cent*" OR "urgent care cent*" OR "urgent care clinic*" OR "care delivery setting*" OR "care facilit*" OR "child guidance clinic*" )

## References

[1] World Health Organization. Noncommunicable diseases. Accessed January 18, 2025. https://www.who.int/news-room/fact-sheets/detail/noncommunicable-diseases

[2] Watson KB , Wiltz JL , Nhim K , Kaufmann RB , Thomas CW , Greenlund KJ . Trends in multiple chronic conditions among US adults, by life stage, Behavioral Risk Factor Surveillance System, 2013–2023. *Prev Chronic Dis.* 2025;22:E15. 10.5888/pcd22.240539 40245168 PMC12007472

[3] Bauer UE , Briss PA , Goodman RA , Bowman BA . Prevention of chronic disease in the 21st century: elimination of the leading preventable causes of premature death and disability in the USA. *Lancet.* 2014;384(9937):45–52. 10.1016/S0140-6736(14)60648-6 24996589

[4] Sung M , He J , Zhou Q , Chen Y , Ji JS , Chen H , . Using an integrated framework to investigate the facilitators and barriers of health information technology implementation in noncommunicable disease management: systematic review. *J Med Internet Res.* 2022;24(7):e37338. 10.2196/37338 35857364 PMC9350822

[5] Food and Drug Administration. What is digital health? 2020. Accessed September 2, 2026. https://www.fda.gov/medical-devices/digital-health-center-excellence/what-digital-health

[6] Bashi N , Karunanithi M , Fatehi F , Ding H , Walters D . Remote monitoring of patients with heart failure: an overview of systematic reviews. *J Med Internet Res.* 2017;19(1):e18. 10.2196/jmir.6571 28108430 PMC5291866

[7] Hanlon P , Daines L , Campbell C , McKinstry B , Weller D , Pinnock H . Telehealth interventions to support self-management of long-term conditions: a systematic metareview of diabetes, heart failure, asthma, chronic obstructive pulmonary disease, and cancer. *J Med Internet Res.* 2017;19(5):e172. 10.2196/jmir.6688 28526671 PMC5451641

[8] Timpel P , Oswald S , Schwarz PEH , Harst L . Mapping the evidence on the effectiveness of telemedicine interventions in diabetes, dyslipidemia, and hypertension: an umbrella review of systematic reviews and meta-analyses. *J Med Internet Res.* 2020;22(3):e16791. 10.2196/16791 32186516 PMC7113804

[9] Hou C , Xu Q , Diao S , Hewitt J , Li J , Carter B . Mobile phone applications and self-management of diabetes: a systematic review with meta-analysis, meta-regression of 21 randomized trials and GRADE. *Diabetes Obes Metab.* 2018;20(8):2009–2013. 10.1111/dom.13307 29582538

[10] Steinhubl SR , Muse ED , Topol EJ . The emerging field of mobile health. *Sci Transl Med.* 2015;7(283):283rv3. 10.1126/scitranslmed.aaa3487 25877894 PMC4748838

[11] Brickwood KJ , Watson G , O’Brien J , Williams AD . Consumer-based wearable activity trackers increase physical activity participation: systematic review and meta-analysis. *JMIR Mhealth Uhealth.* 2019;7(4):e11819. 10.2196/11819 30977740 PMC6484266

[12] Gagnon MP , Ngangue P , Payne-Gagnon J , Desmartis M . m-Health adoption by healthcare professionals: a systematic review. *J Am Med Inform Assoc.* 2016;23(1):212–220. 10.1093/jamia/ocv052 26078410 PMC7814918

[13] Greenhalgh T , Wherton J , Papoutsi C , Lynch J , Hughes G , A’Court C , . Beyond adoption: a new framework for theorizing and evaluating nonadoption, abandonment, and challenges to the scale-up, spread, and sustainability of health and care technologies. *J Med Internet Res.* 2017;19(11):e367. 10.2196/jmir.8775 29092808 PMC5688245

[14] Lee DY , Puri D , Gupta AK , Sonje PD , Lakhanpal S , Singh SK , . From pilots to platforms: making digital health work for chronic disease management. *Front Public Health.* 2026;14:1780035. 10.3389/fpubh.2026.1780035 42254625 PMC13236852

[15] Härkönen H , Lakoma S , Verho A , Torkki P , Leskelä RL , Pennanen P , . Impact of digital services on healthcare and social welfare: an umbrella review. *Int J Nurs Stud.* 2024;152:104692. 10.1016/j.ijnurstu.2024.104692 38301306

[16] Candio P , Ooms T . A conceptual framework and umbrella review of digital health technology interventions and health equity. *BMC Health Serv Res.* 2025;26(1):16. 10.1186/s12913-025-13696-4 41318492 PMC12771798

[17] Powell BJ , Waltz TJ , Chinman MJ , Damschroder LJ , Smith JL , Matthieu MM , . A refined compilation of implementation strategies: results from the Expert Recommendations for Implementing Change (ERIC) project. *Implement Sci.* 2015;10(1):21. 10.1186/s13012-015-0209-1 25889199 PMC4328074

[18] Gates M , Gates A , Pieper D , Fernandes RM , Tricco AC , Moher D , . Reporting guideline for overviews of reviews of healthcare interventions: development of the PRIOR statement. *BMJ.* 2022;378(378):e070849. 10.1136/bmj-2022-070849 35944924 PMC9361065

[19] Belbasis L , Bellou V , Ioannidis JPA . Conducting umbrella reviews. *BMJ Med.* 2022;1(1):e000071. 10.1136/bmjmed-2021-000071 36936579 PMC9951359

[20] Philpot LM , Dugani SB , Singla A , DeZutter M , Ebbert JO . Digital care horizon: a framework for extending health care through digital transformation. *Mayo Clin Proc Digit Health.* 2023;1(3):210–216. 10.1016/j.mcpdig.2023.05.005 37601768 PMC10435276

[21] Damschroder LJ , Reardon CM , Widerquist MAO , Lowery J . The updated Consolidated Framework for Implementation Research based on user feedback. *Implement Sci.* 2022;17(1):75. 10.1186/s13012-022-01245-0 36309746 PMC9617234

[22] Aromataris E , Fernandez R , Godfrey CM , Holly C , Khalil H , Tungpunkom P . Summarizing systematic reviews: methodological development, conduct and reporting of an umbrella review approach. *Int J Evid-Based Health.* 2015;13(3):132–140. 10.1097/XEB.0000000000000055 26360830

[23] Pérez-Bracchiglione J , Meza N , Bangdiwala SI , Niño de Guzmán E , Urrútia G , Bonfill X , . Graphical representation of overlap for OVErviews: GROOVE tool. *Res Synth Methods.* 2022;13(3):381–388. 10.1002/jrsm.1557 35278030

[24] Finnane A , Dallest K , Janda M , Soyer HP . Teledermatology for the diagnosis and management of skin cancer: a systematic review. *JAMA Dermatol.* 2017;153(3):319–327. 10.1001/jamadermatol.2016.4361 27926766

[25] Palacholla RS , Fischer N , Coleman A , Agboola S , Kirley K , Felsted J , . Provider- and patient-related barriers to and facilitators of digital health technology adoption for hypertension management: scoping review. *JMIR Cardio.* 2019;3(1):e11951. 10.2196/11951 31758771 PMC6834226

[26] Helleman J , Kruitwagen ET , van den Berg LH , Visser-Meily JMA , Beelen A . The current use of telehealth in ALS care and the barriers to and facilitators of implementation: a systematic review. *Amyotroph Lateral Scler Frontotemporal Degener.* 2020;21(3-4):167–182. 10.1080/21678421.2019.1706581 31878794

[27] Foong HF , Myint B , Zee K , Car LT . Facilitators and barriers of using digital technology for the management of diabetic foot ulcers: a qualitative systematic review. 2020;17(5):1266–1281. 32390305 10.1111/iwj.13396 PMC7948580

[28] Clement J , Maldonado AQ . Augmenting the transplant team with artificial intelligence: toward meaningful AI use in solid organ transplant. *Front Immunol.* 2021;12:694222. 10.3389/fimmu.2021.694222 34177958 PMC8226178

[29] Hopstaken JS , Verweij L , van Laarhoven CJHM , Blijlevens NMA , Stommel MWJ , Hermens RPMG . Effect of digital care platforms on quality of care for oncological patients and barriers and facilitators for their implementation: systematic review. *J Med Internet Res.* 2021;23(9):e28869. 10.2196/28869 34559057 PMC8501408

[30] Whitelaw S , Pellegrini DM , Mamas MA , Cowie M , Van Spall HGC . Barriers and facilitators of the uptake of digital health technology in cardiovascular care: a systematic scoping review. *Eur Heart J Digit Health.* 2021;2(1):62–74. 10.1093/ehjdh/ztab005 34048508 PMC8139413

[31] Bamgboje-Ayodele A , Borg DN , Mcphail SM , Baysari MT . Clinical decision support systems for chronic obstructive pulmonary disease (COPD) in hospitals: a systematic review. *Digit Health.* 2023;9:20552076231219107. 10.1177/20552076231219107 38089165 PMC10714883

[32] Ramachandran HJ , Oh JL , Cheong YK , Jiang Y , Teo JYC , Seah CWA , . Barriers and facilitators to the adoption of digital health interventions for COPD management: a scoping review. *Heart Lung.* 2023;59:117–127. 10.1016/j.hrtlng.2023.02.004 36801546

[33] Rosenstrom S , Groth S , Risom SS , Hove JD , Brodsgaard A . Nurses’ experiences with virtual consultations and home-monitoring in patients with cardiac disease: a systematic review and qualitative meta-synthesis of results. *Nurs Health Sci.* 2024;26(4):e13180. 10.1111/nhs.13180 39437992

[34] Goh S , Goh RSJ , Chong B , Ng QX , Koh GCH , Ngiam KY , . Challenges in implementing artificial intelligence in breast cancer screening programs: systematic review and framework for safe adoption. *J Med Internet Res.* 2025;27:e62941. 10.2196/62941 40373301 PMC12123233

[35] Kissi J , Annobil C . The advantageous impact of telestroke: global insights and implications for Africa: a scoping review of literature. *Stroke Res Treat.* 2025;2025(1):5635369. 10.1155/srat/5635369 40161482 PMC11949608

[36] Naous E , Achkar A , Morin RA , Mitri J . A systematic review on the impact of electronic consultations on diabetes care. *Endocr Pract.* 2025;31(6):829–838. 10.1016/j.eprac.2025.03.001 40057305

[37] Salmi EM , Basile FW , Khan FA , Watt L , Song R , Bijker EM . Facilitators and barriers affecting the implementation of e-health for chronic respiratory diseases in remote settings: a qualitative evidence synthesis. *BMC Health Serv Res.* 2025;25(1):19. 10.1186/s12913-024-12050-4 39754241 PMC11699737

[38] Grol R , Wensing M . What drives change? Barriers to and incentives for achieving evidence-based practice. *Med J Aust.* 2004;180(S6):S57–S60. 10.5694/j.1326-5377.2004.tb05948.x 15012583

[39] Ferlie EB , Shortell SM . Improving the quality of health care in the United Kingdom and the United States: a framework for change. *Milbank Q.* 2001;79(2):281–315. 10.1111/1468-0009.00206 11439467 PMC2751188

[40] Glasgow RE , Harden SM , Gaglio B , Rabin B , Smith ML , Porter GC , . RE-AIM Planning and evaluation framework: adapting to new science and practice with a 20-year review. *Front Public Health.* 2019;7(64):64. 10.3389/fpubh.2019.00064 30984733 PMC6450067

[41] Booth A , Noyes J , Flemming K , Gerhardus A , Wahlster P , van der Wilt GJ , . Structured methodology review identified seven (RETREAT) criteria for selecting qualitative evidence synthesis approaches. *J Clin Epidemiol.* 2018;99(99):41–52. 10.1016/j.jclinepi.2018.03.003 29548841

[42] Richardson S , Lawrence K , Schoenthaler AM , Mann D . A framework for digital health equity. *NPJ Digit Med.* 2022;5(1):119. 10.1038/s41746-022-00663-0 35982146 PMC9387425

[43] Graham AK , Lattie EG , Powell BJ , Lyon AR , Smith JD , Schueller SM , . Implementation strategies for digital mental health interventions in health care settings. *Am Psychol.* 2020;75(8):1080–1092. 10.1037/amp0000686 33252946 PMC7709140

[44] Pearce L , Costa N , Sherrington C , Hassett L . Implementation of digital health interventions in rehabilitation: a scoping review. *Clin Rehabil.* 2023;37(11):1533–1551. 10.1177/02692155231172299 37132030

[45] Murtagh S , McCombe G , Broughan J , Carroll Á , Casey M , Harrold Á , . Integrating primary and secondary care to enhance chronic disease management: a scoping review. *Int J Integr Care.* 2021;21(1):4. 10.5334/ijic.5508 33613136 PMC7880002

[46] Benavidez GA , Zahnd WE , Hung P , Eberth JM . Chronic disease prevalence in the US: sociodemographic and geographic variations by zip code tabulation area. *Prev Chronic Dis.* 2024;21:E14. 10.5888/pcd21.230267 38426538 PMC10944638

[47] Ljungholm L , Edin-Liljegren A , Ekstedt M , Klinga C . What is needed for continuity of care and how can we achieve it? Perceptions among multiprofessionals on the chronic care trajectory. *BMC Health Serv Res.* 2022;22(1):686. 10.1186/s12913-022-08023-0 35606787 PMC9125858

[48] Demirci D , Minhas MH , Lokker C , Demers C . Primary care physicians’ perspectives on digital health tools for chronic disease management: a rapid review. *PLOS Digit Health.* 2025;4(11):e0001085. 10.1371/journal.pdig.0001085 41264602 PMC12633909

[49] CFIR Research Team, Center for Clinical Management Research. CFIR-ERIC implementation strategy matching tool. Accessed January 18, 2025. https://cfirguide.org/choosing-strategies

